# The Implicit Contribution of Fine Motor Skills to Mathematical Insight in Early Childhood

**DOI:** 10.3389/fpsyg.2020.01143

**Published:** 2020-06-03

**Authors:** Ursula Fischer, Sebastian P. Suggate, Heidrun Stoeger

**Affiliations:** ^1^Department of Sport Science, University of Konstanz, Konstanz, Germany; ^2^Thurgau University of Teacher Education, Kreuzlingen, Switzerland; ^3^Department of Educational Science, University of Regensburg, Regensburg, Germany

**Keywords:** fine motor skills, dexterity, graphomotor skills, finger counting, numerical skills, embodied numerosity, early mathematics

## Abstract

Understanding number magnitude is an important prerequisite for children’s mathematical development. One early experience that contributes to this understanding is the common practice of finger counting. Recent research suggested that through repeated finger counting, children internalize their fingers as representations of number magnitude. Furthermore, finger counting habits have been proposed to predict concurrent and future mathematical performance. However, little is known about how finger-based number representations are formed and by which processes they could influence mathematical development. Regarding the emergence of finger-based number representations, it is likely that they result from repeated practice of finger counting. Accordingly, children need sufficient fine motor skills (FMS) to successfully count on their fingers. However, the role that different types of FMS (such as dexterity and graphomotor skills) might play in the development of finger-based number representations is still unknown. In the current study, we investigated (a) whether children’s FMS (dexterity and graphomotor skills) are associated with their emerging finger-based number representations (ordinal and cardinal), (b) whether FMS explain variance in children’s finger-based number representations beyond the influence of general cognitive skills, and (c) whether the association between FMS and numerical skills is mediated by finger-based representations. We tested associations between preschool children’s (*N* = 80) FMS (dexterity and graphomotor skills), finger-based number representations, and numerical skills. Furthermore, visuo-spatial working memory and nonverbal intelligence were controlled for. Dexterity was related to children’s finger-based number representations as well as numerical skills after controlling for chronological age, but not after also controlling for cognitive skills. Moreover, the relationship between dexterity and numerical skills was mediated by finger-based number representations. No such associations were observed for graphomotor skills. These results suggest that dexterity plays a role in children’s development of finger-based number representations, which in turn contribute to their numerical skills. Possible explanations are discussed.

## Introduction

Early in development, children learn implicitly about numerical and mathematical constructs. Even before the beginning of formal instruction, children have their first experiences of magnitude, through enumerations and comparisons (Geary, 2000). Specifically, children are able to discriminate between different amounts, quantities, or magnitudes, perhaps by virtue of possessing what has been referred to as an innate “number sense” (e.g., Dehaene, 1997). As early as six months of age, children have been reported to be capable of discriminating between sets of objects or sequences of sounds that differ in numerosity by a large enough ratio (Xu and Spelke, 2000; Xu et al., 2005). For example, when presented with two auditory sequences, they notice the difference between 8 and 16 sounds, but not between 8 and 12 sounds. This ability improves across early development, with nine month old infants being able to discriminate 8 from 12 dots, but not 8 from 10 [for an overview see Lipton and Spelke (2004)].

Accordingly, children learn their first number words at the age of two years (Wynn, 1992). At this age, they are also capable of rapidly and accurately recognizing the numerosity of small sets of 1-3 objects without counting; a process also referred to as “subitizing” (Kaufman et al., 1949; Hannula et al., 2007). Their numerical abilities then develop further through interactions with the world and experiences with numerical activities, so that children enter school with a surprising amount of what Baroody and Wilkins (1999) called “informal mathematical knowledge” (p. 84).

Especially in these early stages of informal learning, children’s hands play an important role in their interactions with the world. For example, they use their hands to explore and manipulate objects (e.g., Śniegulska and Pisula, 2013). Further, fingers are used when children first conceptualize numerical magnitude, also referred to as numerosity (e.g., Butterworth, 1999). They use their hands to touch objects during counting, but they also use their fingers as a counting aid when learning about number words and remembering the counting sequence (Lafay et al., 2013). As an important basic numerical skill, counting is strongly associated with children’s development of mathematical skills later in life (Pixner et al., 2017).

However, not only basic numerical skills, such as counting, contribute to the development of mathematical skills. Domain general abilities such as fine motor skills (FMS) have also received increasing research interest due to their association with children’s mathematical abilities (Luo et al., 2007; Pitchford et al., 2016). FMS can be defined as “small muscle movements requiring close eye-hand coordination” (Luo et al., 2007, p. 596). However, the working mechanisms by which FMS are associated with mathematical skills are still largely unresearched.

Based on recent findings, we suggest that one possible mechanism by which the association between FMS and mathematical skills could be formed is the procedure of finger counting. We argue that by internalizing and automatizing repeated finger counting procedures, children come to represent numbers as finger patterns. These finger-based representations of number might then form a stable association between finger movements and numerical content (Roesch and Moeller, 2015).

We therefore start by giving an overview of associations between FMS and mathematics, before describing the development of finger-based representations and their implications for mathematical learning. Finally, we present a working model on how FMS and finger-based representations might interact to contribute to the acquisition of numerical and mathematical knowledge, which formed the basis for the current study.

### Fine Motor Skills and Mathematical Skills

A growing number of studies suggest that children’s FMS are linked to their mathematical skills (Luo et al., 2007; Roebers et al., 2014; Pitchford et al., 2016; Suggate et al., 2017; Fischer et al., 2018a). Especially in school, children with good FMS display better mathematical performance than their peers with lower FMS.

However, explanations for these findings are sparse and have for the most part been very general. Some of these explanations posit that the association is not specific, but that executive functions or general cognitive skills underlie performance in both FMS and mathematics. For example, growing proficiency in writing/graphomotor skills has been hypothesized to free up working memory capacities for mathematical tasks (Luo et al., 2007). Indeed, many studies have shown that working memory capacity is a relevant predictor for mathematical abilities (Alloway and Passolunghi, 2011; Geary et al., 2013; Li and Geary, 2013), with especially visuo-spatial working memory predicting mathematical outcomes longitudinally. For example, Bull et al. (2008) found that preschoolers’ backward visuo-spatial memory span in a Corsi Block Tapping task significantly predicted their mathematical ability three years later. It has been argued that this association exists because visuo-spatial working memory “functions as a mental blackboard to support number representation, such as place value and alignment in columns, in counting and arithmetic tasks” (Alloway and Passolunghi, 2011, p. 133).

Likewise, verbal working memory has been found to be associated with FMS (i.e., visuomotor skills) as measured with a figure copying task (Becker et al., 2014). However, in the same study, Becker et al. (2014) found that although visuomotor skills were related to mathematical skills, verbal working memory was not. Results such as these imply that visuo-spatial working memory is especially relevant for mathematics performance (Alloway and Passolunghi, 2011), although some studies suggest that verbal working memory becomes more relevant with age (Rasmussen and Bisanz, 2005).

Another common factor hypothesised to underlie the association between both FMS and mathematics are general cognitive abilities (Luo et al., 2007; see also Carlson et al., 2013). General cognitive abilities (i.e., intelligence) play an important role in children’s academic development in more aspects than just mathematics, with research indicating that reading and mathematical skills are influenced to the same degree by intelligence (Schneider and Niklas, 2017). Although working memory has been suggested to be a stronger predictor of academic achievement by some (Alloway and Alloway, 2010), others have reported that in early development, intelligence has a greater impact (Schneider and Niklas, 2017). For mathematics achievement specifically, nonverbal intelligence has repeatedly been identified as predictive (e.g., Manolitsis et al., 2013; Hassinger-Das et al., 2014). Turning to FMS, Davis and colleagues (Davis et al., 2011) found that within the construct of general intelligence, especially visual processing was associated with FMS.

In some of the most recent works, however, it has been argued that the missing link for why an association between FMS and mathematical skills exists might lie in children’s early counting experiences (Suggate et al., 2017; Fischer et al., 2018a). Among all the numerical abilities acquired in early childhood, the mastery of the counting procedure has probably received the greatest research interest (e.g., Gelman and Gallistel, 1978; Wynn, 1992; Dowker, 2008; Colomé and Noël, 2012; Aunio and Räsänen, 2015; Fischer et al., 2018a). One reason for this attention could lie in the high predictive value of children’s counting skills for their later mathematical abilities (e.g., Greeno et al., 1984; Stock et al., 2009; Koponen et al., 2016; Mercader et al., 2018). Therefore, the acquisition of counting skills is well-documented, as is the involvement of fingers in attaining this developmental milestone.

Described by Ifrah (1998) as the ‘earliest calculating machine’, fingers have long been used to aid counting and calculation. Numerous studies suggest that finger use in early counting is almost universal (Butterworth, 1999; Lafay et al., 2013; Crollen and Noël, 2015). As such, finger counting has been suggested to be a necessary step in numerical development (Moeller et al., 2011), or at least a helpful tool for numerical development (Lafay et al., 2013). Finger use not only supports children in learning to count, but might also help them to develop conceptual understanding of the purpose of the counting procedure (Siegler, 1991), and thus, the meaning of numbers (Domahs et al., 2008; Fischer, 2008; Fischer et al., 2018a). Interestingly, the use of fingers for counting and calculating is often prohibited or at least frowned upon in schools, most likely because it is considered an immature strategy that should be replaced early on with more abstract representations of number (Moeller et al., 2011). Furthermore, children with mathematical learning difficulties (or dyscalculia) are often reported to remain active finger counters for much longer than their peers (Geary et al., 2004). However, this might simply be due to these children not progressing from counting strategies to the retrieval of memorized arithmetic facts, rather than being a problem of the use of fingers per se (Geary et al., 2004). Accordingly, the current state of research indicates that the use of fingers for calculation might actually help rather than impede children’s mathematical development (e.g., Kaufmann, 2008; for a discussion see Moeller et al., 2011).

Accordingly, research on associations between FMS and mathematical skills has increasingly focused on counting and finger counting. Stronger links have been observed between children’s finger FMS and their performance on finger-based mathematical tasks, such as finger counting and finger calculation, compared to their performance on non-finger-based tasks, such as object counting and verbal calculation (Suggate et al., 2017). Furthermore, the association seems to be driven by the finger counting procedure rather than the outcome. In a recent study involving German preschool children, Fischer et al. (2018a) observed that FMS were related to children’s procedural counting skills (such as correctly assigning one number word to each counted object), which in turn influenced their conceptual understanding of counting (such as understanding that the last number in the counting sequence represents the numerosity of the counted set). Accordingly, these previous results suggest that FMS are particularly relevant for children to acquire proficiency in correctly counting and that understanding the purpose of the counting procedure seems to result from this increase in counting proficiency.

However, not all aspects of FMS might be equally relevant for children’s acquisition of counting skills. As FMS consist of multiple facets, there might be some aspects that are more strongly associated with mathematical development than others. Generally, previous research suggests that not just for mathematics, but also for other cognitive skills, different facets of FMS are relevant to varying degrees (Suggate et al., 2016; Martzog et al., 2019; Fischer et al., 2018b). Specifically, some of the most recent studies on the association between FMS and numerical skills employed dexterity measures, that is, measures that require precise object manipulation skills (Suggate et al., 2017; Fischer et al., 2018a). However, other facets such as graphomotor or visuomotor skills (i.e., tasks that are performed with a pencil) were not considered, although they are found to be associated with mathematics achievement in elementary school children.

To date, in terms of kindergarten children, only one study in particular differentiated between graphomotor skills and another facet of FMS, specifically finger agility (i.e., tasks that require the ability to move one’s fingers independently, see also Butterworth, 1999). In this study with children who attended the last year of kindergarten, Roesch and colleagues (unpublished study reported in a summative article by Fischer et al., 2018b) investigated associations between graphomotor skills, finger agility, and early calculation skills. In contrast to previous studies, in which finger agility was often operationalized as speeded tapping movements with a single finger (e.g., Penner-Wilger et al., 2007), it was here operationalized as deliberate taps with different fingers without time constraints. The authors found that only finger agility, but not graphomotor skills predicted children’s early calculation skills. One possible explanation for this finding was that the deliberate movement of single fingers is necessary for children’s early finger counting activities, as previously suggested by Butterworth (1999). Likewise, previous observations of associations between dexterity and numerical skills might stem from children either manipulating countable objects or their own fingers with their hands during counting activities. Accordingly, based on this previous research, graphomotor skills might not be relevant for children’s early numerical development, whereas other facets of FMS such as finger agility and dexterity might be. They might however become more important when mathematical skills are taught in school and numbers are interacted with in a written format.

### Internalizing Finger-Based Number Representations Through Counting

Finger counting routines are learned by children observing and imitating others’ behavior (Fuson, 1988; Andres and Pesenti, 2015) in a manner typical of a specific culture. Crucially, because these cultural conventions for finger counting are stable within a given culture (i.e., in German finger counting, counting always starts with the thumb for “one”), certain fingers are almost always associated with the same number word during finger counting. This is why it has been suggested that early finger counting experiences lead children to internalize fingers as implicit representations of numbers, in which certain finger constellations are consistently associated with a specific magnitude (Lafay et al., 2013; Adriano et al., 2014; Wasner et al., 2015).

There are different formats in which numbers are mentally represented other than as finger constellations. According to models of numerical processing such as the triple-code model by Dehaene and colleagues (Dehaene, 1992; Dehaene and Cohen, 1995), there are three codes in which humans represent number. The model suggests that adults represent numbers verbally as spoken number words, visually as Arabic numerals, and amodally as magnitudes along a mental number line. In addition to these three codes, Roesch and Moeller (2015) suggested that finger-based representations can be viewed as another representational format of numbers (Roesch and Moeller, 2015). These finger-based representations have been hypothesized to exist in two different forms, the first being an ordinal representation and therefore representing the finger counting process; and the second representing actual cardinal magnitudes rather than a counting sequence in finger-based pictorial form (Wasner et al., 2015). Regarding the order of acquisition of these finger-based representations, researchers argue that ordinal representations are likely acquired before cardinal representations (Roesch and Moeller, 2015; Wasner et al., 2015). However, the literature on the general development of ordinality and cardinality understanding is inconclusive on this issue. Although some have reported that ordinality precedes cardinality (e.g., Siegler, 1991; Bermejo, 1996), later studies find that the development might not be sequential or hierarchical, instead suggesting an iterative development in which both concepts develop in parallel (Rittle-Johnson et al., 2001). One study by Colomé and Noël (Colomé and Noël, 2012) even presents results supporting the opposite view, with children seemingly mastering cardinality before ordinality.

So although the development of these finger-based number representations is not yet fully understood, it is well-established that these representations are permanent. Interestingly, evidence for stable finger-based representations of numbers has been observed not only in children, but also in adult participants (Domahs et al., 2008; Domahs et al., 2010). In these first studies investigating the pervasive influence of finger counting on mathematical cognition, finger-based representations were indirectly measured by assessing how often participants erred by five in arithmetic tasks (Domahs et al., 2008; Klein et al., 2011). The inference of these studies was that errors that deviate by five from the correct result are caused by participants representing numbers in multiples of five, due to their reliance on finger-based representations. Thus, finding that errors of ± 5 were more frequent than errors of ± 4 from the correct result was interpreted to originate from a subconscious activation of finger-based representations (i.e., erring by one hand). However, directly assessing how finger-based representations develop and are associated with numerical skills in early childhood could give further insight into how and when these representations are meaningful for development.

### The Current Study

Although previous research has hinted at a possible link between FMS and mathematical skills via finger counting experiences (Suggate et al., 2017; Fischer et al., 2018a), this link has not been tested directly. Although Wasner et al. (2015) suggested that motor constraints might play a role in the development of finger-based numerical representations, no data exist to directly confirm this association.

In this study, we therefore investigated in depth how two types of finger-based number representations (ordinal and cardinal) interact with FMS and numerical skills. Furthermore, building on previous research suggesting different associations based on different facets of FMS (e.g., Fischer et al., 2018b), we measured FMS using both tasks geared more toward measuring finger dexterity in a classical sense as well as a task assessing graphomotor skill via drawing in a line tracing paradigm. Because children’s early counting experiences rarely involve writing or drawing, but might require finger agility and dexterity, this distinction seems paramount when investigating the genesis of finger-based number representations. Accordingly, we differentiate for the first time both between different types of finger-based number representations (ordinal and cardinal) as well as different types of FMS (dexterity and graphomotor skills).

**FIGURE 1:**
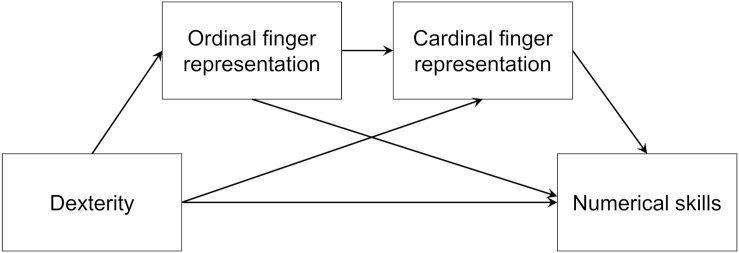
Hypothetical mediation between dexterity and numerical skills via finger-based number representations.

In a correlational design, we tested preschool children on their finger dexterity, graphomotor skill, ordinal and cardinal finger-based representations, and numerical skills. We expected that both children’s ordinal and cardinal finger-based numerical representations should be associated with their dexterity, but not graphomotor skill. Furthermore, we expected that their numerical skills should be associated with their dexterity but not their graphomotor skill. Building on the previously untested hypothesis that finger counting could be the missing link between FMS and mathematical skills, we expected that finger-based number representations would mediate the association between dexterity and numerical skills (see Figure 1). Based on previous theoretical work that suggests that ordinal finger-based representations might be acquired at an earlier developmental stage than cardinal finger-based representations (c.f. Roesch and Moeller, 2015), ordinal finger representations were placed before cardinal finger representations in the model. This should not however imply that cardinal finger representations develop hierarchically from ordinal finger representations.

In addition to investigating these associations, we controlled for maturation, nonverbal intelligence, and visuo-spatial working memory. Thereby, we wanted to account for alternative explanations of the observed associations.

Specifically, we hypothesized that: (a) Dexterity, but not graphomotor skill, is associated with ordinal and cardinal finger-based representations; (b) Dexterity, but not graphomotor skill, is significantly related to both types of finger-based representations, even when controlling for age and other cognitive skills; and (c) the association between dexterity and numerical skills is mediated by finger-based number representations.

## Materials and Methods

### Ethics Approval Statement

This study was carried out in accordance with the recommendations of the Ethical Principles of the German Psychological Society (DGP) and the Association of German Professional Psychologists (BDP). Written informed parental consent was obtained and children gave their verbal assent prior to test administration, in accordance with the Declaration of Helsinki.

### Participants

Prior to testing, we conducted an *a priori* power analysis to determine the necessary number of participants using the program G^∗^Power 3.1.9.2 (Faul et al., 2009). We assumed a medium effect size of around *f* = 0.20 for our mediation model and strived to acquire sufficient statistical power of 0.85. Accordingly, for a multiple regression with four predictors in the final model, the power analysis suggested a sample size of at least 73 participants.

Eighty-two German preschool children attending public German kindergartens participated in the study. Because two children (both boys) did not complete either one or both sessions, they were excluded from the analysis due to incomplete data. This resulted in a final sample of 80 children (40 boys; age: *M* = 4;8 years, *SD* = 11 months, range: 3;1 – 6;3 years). By year, the sample consisted of 19 three-year-olds, 28 four-year-olds, 27 five-year-olds, and 6 six-year-olds. According to a parent questionnaire, which was handed out to the parents together with the consent forms, 11.3% of children spoke a language other than German at home, and 6.3% of children were born outside of Germany. Also, 43.8% of parents reported having a university degree, which is substantially higher than the national average of around 29% (OECD, 2018).

### Test Battery

#### Finger-Based Number Representations

To assess children’s ordinal and cardinal finger-based number representations, two types of finger-based tasks were administered (comparable to the ordinal and cardinal tasks used by Wasner et al., 2015). Ordinal finger-based number representations were assessed using a finger counting paradigm, whereas cardinal finger-based number representations were assessed using a paradigm in which children were asked to show a number (i.e., finger montring).

##### Ordinal finger-based representation: finger counting

In the finger counting task, which assessed children’s ordinal finger-based number representation, children were asked to count on their fingers to a given number (e.g., “Please count to four on your fingers.”). All numbers from 1–10 were administered in a pseudo-randomized order: Numbers 1–5 were presented prior to numbers 6–10, as the latter needed to be counted on both hands and switching between one and two hands could have been confusing or too difficult for the younger children in our sample. The experimenter documented the precise order in which the child extended his or her fingers as well as whether the verbal counting sequence was recited correctly, with one number word uttered per extended finger. A trial counted as solved if the child both correctly counted verbally and extended one finger per number word, and the counting resulted in the correct number of extended digits. Which fingers children used did not play a role in the scoring, so children could, for example, start counting with their right or left hand as well as with their pinkie finger or thumb. Children could score a maximum of 10 points in this task.

##### Cardinal finger-based representation: finger montring

In the finger montring task, children’s cardinal finger-based number representation was assessed. To this end, children were asked to show a certain number with their fingers (e.g., “Please show me four fingers.”). Again, numbers 1–5 were presented prior to numbers 6–10 in a pseudo-randomized order. The experimenter documented which fingers the child extended and whether he or she extended the correct amount of fingers, and also noted whether children extended their fingers simultaneously or consecutively. Because this task was supposed to measure whether children had internalized number magnitudes as finger patterns, a trial only counted as solved if the child extended the fingers simultaneously without counting. Again, the fingers that children used to display each number was not relevant for the scoring. The maximum score was 10 points.

#### Numerical Tasks

In order to test for the direct influence of both FMS as well as finger-based numerical representations on mathematical skills, we included additional numerical tasks that were not related to finger use. These tasks were chosen to cover the different formats in which numbers can be represented (non-symbolic as concrete magnitudes, visually as Arabic digits, and verbally as number words in the counting sequence), which also correspond to the first three steps of numerical development according to the “Four-step-developmental model of numerical cognition” described by von Aster and Shalev (von Aster and Shalev, 2007). In this model, children acquire understanding of concrete magnitude in infancy, followed by number words in their preschool years, and Arabic digits upon entering school. Accordingly, when combined into a composite score, the varying levels of difficulty of these tasks should allow for an accurate assessment of children’s numerical skills, even given the large age range of our sample. To test whether the tasks measure a common underlying construct that can be combined into a composite score, we entered them into a principal component analysis. This analysis revealed that the three tasks loaded on one unitary factor, which explained 83.0% of the variance in numerical skills, therefore supporting our decision to use a composite score.

##### Non-symbolic dot comparison

We measured children’s ability to compare non-symbolic magnitudes by means of the numeracy screener (Nosworthy et al., 2013), in which children are required to compare two dot patterns and determine which of two dot patterns contains more dots than the other. This task is timed, so that children are given 120 s to solve as many of the 56 comparisons as possible. Although this task is generally paper-pencil, we adapted it to our young participants and therefore printed it out in double size and had children point at the more numerous array rather than cross it out. This way, graphomotor requirements of the task were minimized as well. The experimenter instead checked on an answer sheet which array the children pointed at. The sum of correctly solved items within the time limit was used in the analysis, with possible scores ranging from 0 to 56. Test-retest reliability for this task after three weeks was previously reported to be *r* = 0.62, and convergent validity with a computerized non-symbolic comparison task was reported to be *r* = 0.30 (Nosworthy, 2013).

##### Symbolic number comparison

The same paradigm was presented a second time in a symbolic version, which was also adapted from the numeracy screener (Nosworthy et al., 2013). Children had to indicate which of two Arabic digits was larger than the other by pointing at it, and the experimenter checked the response on the answer sheet. Although many participants struggled with the task, most of them were familiar with small numbers such as 1 and 2, and accordingly solved more than 50% of the items they attempted correctly. Again, children were asked to solve as many of the 56 comparisons as possible within 120 s. The sum of items they solved correctly was used in the analysis, with possible scores ranging from 0 to 56. The reported test-retest reliability for this task after three weeks was *r* = 0.67, and convergent validity with a computerized symbolic comparison task was *r* = 0.61 (Nosworthy, 2013).

##### Verbal counting sequence

In order to assess whether children were familiar with number words, we tested their knowledge of the verbal counting sequence without the additional requirement of counting objects or fingers. In this task, children were asked to simply count aloud as far as they could. In accordance with instructions given in the standardized test battery TEDI-MATH (Kaufmann et al., 2009), children were given help with starting the sequence if they did not know what to do (“Count like this: one, two, and now you!”) and were stopped at the number 31 if they did get that far. We scored the largest number the child counted correctly before making a mistake. For example, if a child counted ‘1, 2, 3, 5, 6…’, the score would be ‘3’. The maximum score in this task was 31, and was determined by the cut-off criterion.

#### Fine Motor Skills

To test children’s dexterity and graphomotor skills, the manual dexterity scale of the Movement-ABC 2 (M-ABC 2, Petermann, 2015) was administered. This scale consists of three tasks, two of which were categorized as measuring dexterity (coin posting and bead threading), while the third (Drawing trail) was used as an indicator for graphomotor skills.

##### Dexterity

###### Coin posting

Children were asked to insert coins into a slot in a box as quickly as they could. Children from 3-4 years old received 6 coins, whereas children aged 5-6 years received 12 coins. Children were encouraged to use their dominant hand for this task, and were given two trials, the faster of which was scored. To make the scores for 3-4 and 5-6 year-olds comparable, these scores where converted into standardized scores according to the M-ABC 2 manual, which were then used in the analysis. Excellent test-retest reliability after one week was reported for this task in a Greek study, ICC = 0.93 (Ellinoudis et al., 2011).

###### Bead threading

In the bead-threading task, children were instructed to thread square beads onto a string with a pointed end that made the beading easier. Again, children aged 3-4 years received 6 beads, and children aged 5-6 years received 12 beads. The beads were placed in a line in front of them and children were again instructed to complete the task as fast as possible. Out of two trials, the faster was scored. As in the coin posting task, the time children needed to complete the fastest trial was transformed into standardized scores using the M-ABC 2 manual. Test-retest reliability for this task was also reported to be excellent, ICC = 0.92 (Ellinoudis et al., 2011).

##### Graphomotor skills

###### Drawing trail

In the graphomotor portion of the manual dexterity scale, children were presented with a printout of a trail. They were instructed to help a cyclist depicted at the beginning of the trail to reach his house, which was depicted at the end of the trail. Using a red marker, the children had to draw the path for the biker within the boundaries of the trail, preferably without drawing outside the given lines. This procedure was first demonstrated by the experimenter, after which children performed the task twice. Here, children were instructed to work as accurately as possible. The score in this task was calculated by transforming the number of errors children made on the more accurate of the two trials to standardized scores according to the M-ABC 2 manual. For this task, test-retest reliability was reported to be moderate, ICC = 0.66 (Ellinoudis et al., 2011).

#### Control Variables

To control for children’s nonverbal intelligence and visuo-spatial working memory capacity, we administered a subtest from an intelligence test battery (KABC-II, Kaufman and Kaufman, 2015) as well as a visuo-spatial working memory test (Corsi block-tapping task, adapted from Kessels et al., 2000, 2008). According to test norms for the German version, this subtest showed excellent reliability, α_*cr*_ > 0.83.

##### Nonverbal intelligence: conceptual thinking

The conceptual thinking subtest measures children’s ability to reason about classifications of things and objects in a nonverbal format and is part of the problem-solving portion of the KABC-II. In the conceptual thinking subtest, children are presented with 4 or 5 pictures and have to decide which one of the pictures does not fit with the set (e.g., three red umbrellas and one yellow umbrella). Again, children give their response by pointing at the chosen picture and are awarded one point per correct response. In total, the subtest consists of 28 items, but testing stops when a child answers 4 out of 5 consecutive items incorrectly. As for verbal knowledge, a sum score was entered as a covariate in the analysis.

##### Visuo-spatial working memory

Children’s visuo-spatial working memory was assessed via a Corsi block-tapping task, in which children had to memorize and replicate a visually presented sequence. The task was conducted using a wooden board with 9 wooden cubes (3 cm × 3 cm × 3 cm) glued onto it in a non-geometrical pattern (replicated after the layout presented in Kessels et al., 2000). First, the experimenter tapped the cubes in a certain order at a speed of approximately one cube per second. The child was instructed to wait until after the experimenter was finished, and then tap the cubes either in the same (forward span) or reversed (backward span) order. Two items were presented per span length, with difficulty starting at two blocks and increasing up to seven blocks. If the child successfully replicated at least one of the two items of a given length, testing continued with length increasing by one. As soon as two items of the same length were replicated incorrectly, testing was stopped. The longest successfully replicated span – not the number of correctly remembered items – was used in the analysis as the child’s visuo-spatial working memory span for both the forward and backward span.

### Procedure

Parents completed the questionnaire at home and returned it to the kindergarten staff together with the written consent form. Children were then tested individually in their respective kindergartens across two sessions by trained undergraduate students of teaching and the first author. Prior to the beginning of the study, all student testers were familiarized with the procedure and received training by the first author on how to conduct the tests according to the instructions. The first author then conducted the first two testing sessions herself, with the student testers observing. Each tester’s first two testing sessions were conducted under supervision by the first author to ensure that testing procedures were exactly adhered to. The tasks were presented in the same order to each child, and completion of all tasks took approximately 45–60 min per child (two sessions of 20–30 min each).

### Analytical Approach

We first tested whether dexterity was associated with finger-based number representations and numerical skills after controlling for covariates (i.e., age and cognitive skills) via correlation analyses and hierarchical regressions. Secondly, a mediation analysis using a bootstrap sampling method was performed to test the final hypothesis that the association between dexterity and numerical skills was mediated by ordinal and cardinal finger-based number representations. Prior to this analysis, all measures were z-standardized and the analysis was conducted using the PROCESS Macro for SPSS (Hayes, 2013).

In this mediation model, depicted in Figure 1, ordinal finger-based representations were modeled as preceding cardinal finger-based representations, although, research on this developmental path is not fully conclusive. Accordingly, an alternative model with cardinal preceding ordinal finger-based representations was also considered, but did not meet the preconditions for mediation. Notably, ordinal finger-based representations did not have a significant effect on numerical skills in this model.

## Results

### Data Preparation

Because we were specifically interested in associations between finger-based number representations and different facets of FMS, dexterity (i.e., bead threading and coin posting) and graphomotor skill (i.e., drawing trail) were entered separately into the analyses rather than collapsed into a single fine motor score as suggested in the M-ABC 2 manual. The score for dexterity was then calculated as the mean of the bead threading and coin posting scores. Descriptive statistics for the final variables are presented in Table 1.

**TABLE 1:** Descriptive statistics.

	*M*	*SD*	*n*	Min.	Max.	Skew	Kurtosis
Dexterity	10.06	2.64	80	1.00	16.00	–0.57	1.20
Graphomotor skill	9.03	3.32	80	1.00	16.00	–1.08	0.76
Finger-based number representations							
Ordinal	7.32	3.25	78	1.00	10.00	–0.81	–0.95
Cardinal	6.84	3.39	79	0.00	10.00	–0.69	–1.05
Numerical skills (*Z*-score)	0.00	1.00	80	–1.64	1.49	0.07	–1.48
Control variables							
Age (months)	55.56	10.92	80	37.00	75.00	–0.13	–1.05
Visuo-spatial working memory (forward span)	3.30	1.12	80	0.00	5.00	–1.01	1.94
Visuo-spatial working memory (backward span)	2.30	1.74	80	0.00	6.00	0.23	–0.60
Nonverbal intelligence	11.73	5.10	80	0.00	24.00	–0.27	–0.07

### Hypothesis 1: Dexterity, but Not Graphomotor Skill, Is Associated With Ordinal and Cardinal Finger-Based Representations

In a first step, we conducted partial correlations, controlling for chronological age due to the relatively large age span (3;1 – 6;3 years) of our participants. Both raw and partial correlation results are depicted in Table 2.

**TABLE 2:** Pearson correlation coefficients between fine motor skills, finger-based number representations, numerical skills, and control variables.

		1	2	3	4	5	6	7	8
1	Dexterity	–	0.199	0.244*	0.286*	0.268*	−0.075	0.187	0.371**
2	Graphomotor skill	0.207	–	0.018	0.041	0.003	0.001	0.148	−0.007
3	Ordinal finger-based representation	0.150	−0.150	–	0.816**	0.494**	0.146	0.359**	0.317**
4	Cardinal finger-based representation	0.185	−0.139	0.908**	–	0.542**	0.182	0.349**	0.326**
5	Numerical skills	0.185	−0.158	0.751**	0.781**	–	0.216	0.522**	0.545**
6	Working memory forward span	−0.072	−0.121	0.464**	0.487**	0.513**	–	0.274*	0.167
7	Working memory backward span	0.159	−0.004	0.610**	0.604**	0.710**	0.493**	–	0.297**
8	Nonverbal intelligence	0.315**	−0.119	0.563**	0.570**	0.705**	0.403**	0.517**	–
9	Age	0.003	−0.215	0.703**	0.709**	0.728**	0.531**	0.581**	0.539**

Raw correlations are presented below the diagonal and partial correlations controlling for chronological age above the diagonal. * = *p* < 0.05, ** = *p* < 0.01.

#### Correlations of Fine Motor Tasks

As presented in Table 2 above the diagonal, dexterity correlated with ordinal finger-based number representations, *r* = 0.244, *p* < 0.05, cardinal finger-based number representations, *r* = 0.286, *p* < 0.05, numerical skills, *r* = 0.269, *p* < 0.05, and nonverbal intelligence, *r* = 0.371, *p* < 0.01. Children’s graphomotor skills were not significantly correlated with any other variables in the partial correlation analysis.

#### Correlations of Finger-Based Representations

The two types of finger-based representations were highly correlated with each other, *r* = 0.816, *p* < 0.001. In addition to dexterity, both the ordinal and cardinal finger-based representation were significantly correlated with numerical skills, ordinal: *r* = 0.494, *p* < 0.001, cardinal: *r* = 0.542, *p* < 0.001.

Among the control variables, both types of finger-based representations were also significantly correlated with working memory backward span (ordinal: *r* = 0.359, *p* < 0.01, cardinal: *r* = 0.349, *p* < 0.01) and nonverbal intelligence (ordinal: *r* = 0.317, *p* < 0.01, cardinal: *r* = 0.326, *p* < 0.01).

#### Correlations of Numerical Skills

In addition to the above-mentioned correlations, numerical skills were also associated with the working memory backward span, *r* = 0.522, *p* < 0.01, and nonverbal intelligence, *r* = 0.545, *p* < 0.01. Note that children’s working memory forward span was not significantly correlated with any variables of interest.

### Hypothesis 2: Dexterity, but Not Graphomotor Skill, Is Significantly Related to Both Types of Finger-Based Representations, Controlling for Age and Other Cognitive Skills

To test whether dexterity and/or graphomotor skill remained significantly related to finger-based representations when controlling for age and cognitive skills, we conducted hierarchical multiple linear regression analyses. Predicting ordinal and cardinal finger-based representations, we entered dexterity and graphomotor skill in a first step, adding age in the second step. In a third step, we added the control variables visuo-spatial working memory forward and backward span, and nonverbal intelligence. Results for both hierarchical regressions are in Table 3.

**TABLE 3:** Hierarchical linear regression models predicting ordinal and cardinal finger-based representations.

	Ordinal finger-based representation					Cardinal finger-based representation				
Variable	B	SE B	β	*R*^2^	*ρR^2^*	B	SE B	β	*R*^2^	*ρR^2^*
Step 1				0.056	0.056				0.066	0.066
Dexterity	0.232	0.141	0.188			0.287	0.146	0.221		
Graphomotor skill	–0.182	0.111	–0.188			–0.186	0.115	–0.183		
Step 2				0.525**	0.469**				0.546**	0.480**
Dexterity	0.220	0.101	0.178*			0.270	0.103	0.209*		
Graphomotor skill	–0.023	0.081	–0.023			–0.015	0.083	–0.015		
Age in months	0.211	0.025	0.704**			0.224	0.025	0.712**		
Step 3				0.593**	0.068*				0.608***	0.062*
Dexterity	0.124	0.105	0.101			0.185	0.107	0.143		
Graphomotor skill	–0.035	0.078	–0.036			–0.026	0.080	–0.026		
Age in months	0.138	0.032	0.463**			0.149	0.033	0.475**		
Working memory (forward span)	0.128	0.273	0.045			0.262	0.279	0.087		
Working memory (backward span)	0.441	0.189	0.236*			0.397	0.193	0.202*		
Nonverbal intelligence	0.093	0.064	0.146			0.090	0.066	0.134		

* = *p* < 0.05, ** = *p* < 0.01.

#### Predicting Ordinal Finger-Based Representations

For the model predicting ordinal finger-based representations, dexterity and graphomotor skills did not contribute significantly to the model when entered in the first step, *F*(2,76) = 2.243, *p* < 0.05, and explained 5.6% of the variance in ordinal finger-based representations. Adding age to the model significantly increased the explained variance by 46.9%, *F*(1,75) = 27.274, *p* < 0.01, with both dexterity and age, but not graphomotor skills, being significant predictors. When adding the control variables in the third step, explained variance increased by another 6.8%, *F*(3,72) = 17.250, *p* < 0.01. Out of the three control variables, only the visuo-spatial working memory backward span was a significant predictor in this final model. After the control variables were included, dexterity was no longer a significant predictor, whereas the effect of age remained significant.

#### Predicting Cardinal Finger-Based Representations

For cardinal finger-based representations, dexterity and graphomotor skills did not contribute significantly to the model in the first step, *F*(2,76) = 2.702, *p* = 0.073, explaining only 6.6% of the variance. Dexterity was a marginally significant predictor, β = 0.221, *p* = 0.054, whereas graphomotor skill was not, β = −0.183, *p* = 0.110. The explained variance was significantly increased by 48.0%, *F*(1,75) = 30.060, *p* < 0.01, when age was entered in the second step, and both dexterity and age, but not graphomotor skill, were significant predictors. In the third step, adding the control variables increased explained variance by 6.2%, *F*(3,72) = 18.621, *p* < 0.01. Again, visuo-spatial working memory backward span was a significant predictor in this final model. After the control variables were included, age remained a significant predictor, whereas dexterity was no longer a significant predictor, β = 0.143, *p* = 0.088, of cardinal finger-based representations.

### Hypothesis 3: The Association Between Dexterity and Numerical Skills Is Mediated by Finger-Based Number Representations

**FIGURE 2:**
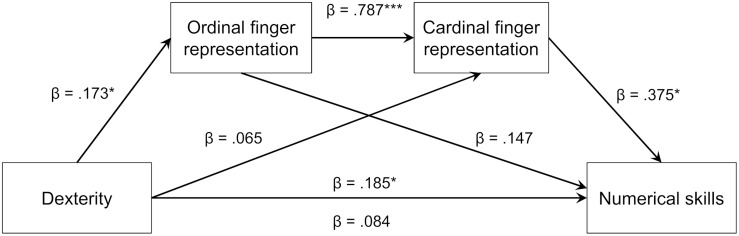
Results of the mediation model for the association between dexterity, finger-based number representations and numerical skills.

For the mediation analysis, we used dexterity as a predictor variable, and both ordinal and cardinal finger representations as mediators to predict numerical skills (see Figure 2). Given that graphomotor skills were neither significantly correlated with numerical skills nor associated with ordinal or cardinal finger-based number representations in the regression analyses, we did not conduct a mediation analysis with graphomotor skill as a predictor. To control for the large age range in our sample, and also because the regression results suggest that age could act as a suppressor for the association between dexterity and finger-based number representations, we controlled for age.

The analysis was conducted using the PROCESS Macro Version 3.3 for SPSS (Hayes, 2013), and was based on 10,000 bootstrap samples using percentile 95% confidence intervals (Preacher and Hayes, 2008). Using bootstrapping methods to estimate confidence intervals was necessary due to the sample size being rather small for a mediation analysis (see e.g., Fritz and MacKinnon, 2007), and in such cases, bootstrapping can provide more accurate inferences (Fox, 2015). It is a method in which repeated samples are drawn from the available data in order to estimate the characteristics of the population (Fox, 2015).

Results confirmed our hypothesis (see Figure 2). Controlling for the effects of age, β = 0.357, *SE* = 0.100, *p* < 0.01, the total effect of children’s finger dexterity on numerical skills, β = 0.185, *SE* = 0.077, *p* < 0.05, was mediated by their finger-based representations as indicated by a significantly reduced, non-significant direct effect after mediation, β = 0.084, *SE* = 0.070, *p* = 0.232. This finding was further corroborated by the significant indirect effect of dexterity on numerical skills via ordinal and cardinal finger-based representations, β = 0.101, *SE* = 0.039, Percentile bootstrap CI [0.033,0.186].

Within this sequential model, two other mediations were observed: The previously significant association between dexterity and cardinal finger-based representations, *r* = 0.286, *p* < 0.05, was fully mediated by ordinal finger-based representations and not significant in the full mediation model. Furthermore, ordinal finger-based representations and numerical skills, which had been significantly correlated before, *r* = 0.494, *p* < 0.01, were no longer significantly associated in the full mediation model (see Figure 2).

## Discussion

In the current study, we investigated for the first time whether FMS are associated with finger-based representations of number, and whether this association might explain the often-observed correlation between FMS and numerical skills (e.g., Luo et al., 2007; Roebers et al., 2014; Pitchford et al., 2016; Suggate et al., 2017; Fischer et al., 2018a). By measuring dexterity, graphomotor skills, finger-based representations of number, and numerical skills, we arrived at a more comprehensive understanding of how these early childhood abilities interact. As the genesis of implicit finger-based representations seems to play a substantial role in children’s numerical development, understanding the underlying working mechanisms was our primary objective.

We observed, as expected, that FMS were associated with finger-based number representations, thereby adding to the growing number of studies findings such links (Luo et al., 2007; Roebers et al., 2014; Pitchford et al., 2016; Suggate et al., 2017; Fischer et al., 2018a). Associations were specific, in that a link was found for dexterity, but not for graphomotor skills. In the hierarchical regressions of ordinal and cardinal finger-based representations, we observed that dexterity explained a small but significant amount of variance in finger-based representations when age was also entered as a predictor. This finding indicates that age might have acted as a suppressor variable, which could be due to the fact that the dexterity tasks were analyzed based on age-normed standard scores, whereas no age-norms were available for the measures of finger-based number representations. Recall that age-norms had to be used for the FMS tasks, as raw scores were not normally distributed. More notably, when control variables were entered into the model in a third step, visuo-spatial working memory backward span explained a significant amount of variance in both types of finger-based representations.

For both ordinal and cardinal finger-based representations, the inclusion of the control variables therefore led to dexterity no longer being significantly related to finger-based representations. It is possible that children’s visuo-spatial working memory plays a larger role in their finger counting / finger montring performance than their FMS. For example, a task analysis of counting and montring would suggest that these require children firstly storing a number concept, secondly finding its corresponding finger-component, and thirdly performing matches between the number concept and fingers. Furthermore, this finding, although not in support of our hypothesis, is consistent with previous research on working memory and finger counting. For example, Dupont-Boime and Thevenot (2018) observed that children with a higher working memory capacity were more likely to spontaneously use their fingers to solve addition problems. These recent findings are in contrast with previous assumptions that children with a lower working memory capacity were more likely to rely on their fingers for finger counting, at least from the middle of primary school (Geary, 1993).

To better understand the unexpected result that dexterity only explained significant variance after including age, we conducted a post-hoc median split for age and repeated the regression analyses for the two resulting age groups (age group 1: 3;0 to 4;8 years, *N* = 39; age group 2: 4;9 to 6;3 years, *N* = 41). If age plays such a pivotal role in the associations between dexterity and finger-based representations, the associations might differ for the two age groups. Indeed, these analyses revealed that for the younger age group, dexterity was a significant predictor for ordinal and cardinal finger-based representations in the first step of the regression, as was originally expected for the entire sample (see Supplementary Table 1). More so, this association remained significant in the second step for cardinal finger-based representations when age was added. In the third step, none of the control variables explained significant variance for either ordinal or cardinal representations.

Results were different for the older age group, for whom no predictors were significant in the first and second step (i.e., FMS and age), and only working memory backward span was a significant predictor in the third step (see Supplementary Table 2). While these post-hoc analyses with reduced sample sizes need to be interpreted with caution, they do hint at a developmental shift in the processes involved in the consolidation of finger-based number representation. Younger children might struggle with the motor demands of finger counting/montring, whereas older children might depend more on retrieving the finger patterns for counting/montring from memory.

Perhaps most importantly, the mediation analysis, in which we tested the assumed association between dexterity, ordinal and cardinal finger-based representations, and numerical skills, supported our hypothesis about how dexterity influences numerical development. The results were consistent with the idea that dexterity might contribute to the development of ordinal and cardinal finger-based representations, which then influence numerical skills.

### Theoretical Implications

The current study contributes to the growing literature on finger counting and finger-based representations, in that it takes a differentiated look at ordinal and cardinal finger-based number representations and their relationship with FMS and domain-general cognitive skills often associated with numerical skills. Our results are in agreement with previous research that suggested that ordinal and cardinal finger-based representations need to be differentiated (Wasner et al., 2015), as they seem to play different roles in children’s numerical skill development. It is also worth noting that children performed slightly worse on the cardinal finger montring task, averaging 6.84 out of 10 points, whereas they averaged 7.32 out of 10 points in ordinal finger counting. However, seeing as this difference was not statistically significant, further research will be necessary to determine whether the development of ordinal finger-based representations really does precede that of cardinal finger-based representations.

Accordingly, the relative contributions of children’s ordinal and cardinal finger-based representations to their numerical skills cannot be deduced from our data. Although cardinality might be more difficult to master and is often considered an important predictor for mathematical development (Geary et al., 2018), it is possible based on previous findings that ordinality might be a more important predictor than cardinality for certain aspects of numerical development. For example, it has been previously suggested that the (spatial) ordering of numbers plays a large part in children’s understanding of symbolic number (i.e., number words and Arabic digits) (Sella et al., 2019). In a similar vein, training children in the ordinal number sequence transfers to their number ordering and number line estimation performance (Xu and LeFevre, 2016). Accordingly, it could be argued that ordinality might be more relevant for some aspects of numerical development in the early stages of child development studied here.

Also, the development of these finger-based number representations seems to rely on different skill sets, with dexterity playing only a minor part compared to maturation effects and the impact of visuo-spatial working memory capacity, as can be seen in the regression analyses. In the future, longitudinal studies should investigate the timeline in which these skills develop and how they influence each other.

At a general level, our findings add to the accumulating body of work pointing to the importance of dexterity as a key FMS in relation to cognitive outcomes. For example, Martzog et al. (2019) found that dexterity was more closely linked to spatial intelligence than hand-eye coordination or repetitive speed-FMS. On the other hand, some work indicates that graphomotor skills play a greater role in reading performance than dexterity does, presumably due to the functional relevance of graphomotor skills to writing and thereby reading (Suggate et al., 2018, 2019).

Especially with regard to the importance of graphomotor skills for mathematics, studies with elementary school children consistently yield associations between the two domains. For example, Pitchford et al. (2016) reported stable associations between a task in which children had to reproduce drawings of geometric shapes and children’s mathematical reasoning skills in first grade. Likewise, Carlson and colleagues (Carlson et al., 2013) observed that in a sample with a broad age range from five to 18 years, participants’ mathematical skills were significantly associated with their performance in tracing and copy-a-figure tasks, which both rely on graphomotor skills. It is therefore possible that when children enter school, their finger use during counting decreases, whereas writing of Arabic digits increases. Thereby, graphomotor skills might gain importance for mathematical learning and performance over time, whereas dexterity may become less relevant. This interpretation is also in agreement with the age-split post-hoc analyses of our data described above. Dexterity seems to play a significant role in children’s finger-based representations up to a certain stage in development, after which other cognitive processes such as working memory take over.

Taken together, the current study adds to the body of work indicating that children’s FMS relate both functionally (i.e., being able to move fingers as a prerequisite to numerical development) and at a representational level to mathematical development (Penner-Wilger and Anderson, 2013). More work such as the current study examining FMS and mathematics in a detailed way is needed.

### Practical Implications for Education and Intervention

The present results highlight the importance in viewing numerical skills in early childhood as a construct influenced by many different facets of children’s cognitive and motor development. Therefore, early childhood professionals and educators should consider children’s FMS as well as their working memory capacity when employing numerical trainings at this early developmental stage. Our results also highlight the positive relationship between children’s finger-based representations and their numerical skills, and thereby adds to previous similar results (e.g., Lafay et al., 2013; Soylu et al., 2018). We therefore argue that fostering children’s early counting skills by encouraging finger use could be beneficial for their later numerical development, and might concurrently train their FMS as well as relieve their working memory load – a notion also suggested by other researchers (e.g., Beller and Bender, 2011).

### Limitations and Future Directions

The current results have given us a first exciting look into how fingers and numbers interact. However, further research will be necessary to delve further into which FMS and numerical skills are specifically associated with finger-based number representations. Notably, only dexterity, but not graphomotor skill was associated with the numerical tasks in the age group surveyed in our study. Although this could also indicate that the Movement-Assessment Battery for Children 2 (Petermann, 2015) might not be the ideal measure for investigating finger-number associations, it is also worth taking a closer look at which tasks did correlate. In both the coin posting and bead threading tasks, children have to move one object after another, either into a box or onto a thread. This sequential moving of objects is very similar to many counting activities in which children move the counted objects from one place to another. Also, the coins represent money, which is also often counted and associated with numbers that indicate its value. It is therefore possible that fine motor tasks that mimic a counting movement are more strongly associated with finger-based representations of number, which also originate in counting movements. In contrast, the trail drawing subtest measures a skill that, at least at the age of children in our study, is not associated with the counting procedure. Future studies should therefore look into fine motor tasks which bear different amounts of resemblance to counting movements.

Another possibility for future studies would be to include tasks that cover additional facets of FMS. For example, while graphomotor and visuomotor skills are often not differentiated (e.g., Mayes et al., 2009; Martzog et al., 2019), there are conceptualizations of FMS that see both as distinct constructs. For example, Becker et al. (2014) differentiate between tasks that require motor control (such as the tracing task in the M-ABC 2) and visuomotor tasks that also require spatial abilities. The most prominent example of a test of visuomotor integration is the Beery-Buktenica Developmental Test of Visual-Motor Integration (Beery VMI; Beery et al., 2010). In this test, participants have to copy figures into a blank square as accurately as possible. Because this task might require more visuo-spatial integration than say, a tracing task, it might be more strongly associated with numerical skills that also have a strong spatial component, such as locating numbers on a number line (e.g., Ebersbach, 2015). This could also explain previous findings of associations between the Beery VMI and mathematical skills (e.g., Simms et al., 2016).

In our study design, we opted for a combined measure of different numerical skills because we were interested in whether finger-based number representations and FMS generally relate to numerical skills. However, this approach did not allow us to investigate associations with specific numerical skills such as knowledge of the verbal counting sequence or of Arabic digits. To investigate whether finger-based number representations are more strongly associated with certain numerical skills (e.g., those that are closely tied to finger counting, such as knowledge of the verbal counting sequence), two changes would be necessary for future studies: Firstly, children of a smaller age range should be tested that are at a comparable skill level in these numerical skills; and secondly, numerical tasks should be used that consist of more items than those in our study and that also measure both accuracy and fluency for a more precise assessment of the respective numerical skills.

With regard to the origins of finger-based number representations, our results suggest that dexterity contributes to their development. However, it is also worth noting that the amount of variance was comparatively small (5–6%), especially in stark contrast to the amount of variance explained by children’s age (47–48%). Here, future research should consider investigating a smaller age range with a similar or even larger sample size than that of our study. Our *post hoc* analyses for different age groups suggest that the association is strongest between the ages of 3 and 4.5 years, so this would be a promising age group for further investigations. It could also be useful to consider children’s general level of development in addition to just their chronological age in future projects.

In the same vein, dexterity only became significantly associated with numerical skills when age was also entered in the analysis, suggesting that age might have acted as a suppressor variable. As noted above, this might have been caused by age-normed standard scores being used for the FMS tasks, but not the finger-based number representation tasks. Accordingly, future studies should consider working with fully unstandardized scores to disentangle the contributions of age and FMS to finger-based number representations.

A promising avenue for future studies investigating finger-based number representations lies in longitudinal designs. Especially when attempting to explain the impact that dexterity has on children’s numerical development via their finger-based number representations, it would be preferable to measure children’s skills at multiple time points in addition to concurrent comparisons.

## Conclusion

In this study, we investigated the link between FMS and children’s early mathematical development, considering children’s finger-based number representations as a potential link between the two. At an age where children use their fingers to interact with numbers and consolidate their finger-based experiences into persistent representations, this is of particular relevance for their mathematical development. Our results highlight that a differentiation between facets of FMS is necessary, as graphomotor skills were not associated with either finger-based number representations or numerical skills. In contrast, links between dexterity, finger-based number representations, and numerical skills were observed; with finger-based number representations mediating the association between dexterity and numerical skills. However, the relationship between dexterity and finger-based number representations was only tentative, depended on children’s age, and was not upheld once visuospatial working memory was controlled for. It seems that the association is stronger for younger children, who rely even more on their fingers to count and depict numerosities. Accordingly, while dexterity might only play a small part in the acquisition of finger-based number representations, this relationship can further our understanding of how dexterity is linked to numerical and mathematical skills.

At a broader level, our findings add to the growing body of work indicating that motor experiences and skills are intimately linked with cognitive skills. Future work is needed to further our understanding of this question of both theoretical and pedagogical significance.

## Data Availability Statement

The datasets generated for this study are available on request to the corresponding author.

## Ethics Statement

Ethical review and approval was not required for the study on human participants in accordance with the local legislation and institutional requirements. Written informed consent to participate in this study was provided by the participants’ legal guardian/next of kin.

## Author Contributions

UF, SS, and HS conceptualized the study, designed the experiment and wrote the manuscript. UF conducted the study, analyzed the data, and wrote a first draft of the manuscript.

## Conflict of Interest

The authors declare that the research was conducted in the absence of any commercial or financial relationships that could be construed as a potential conflict of interest.
