# Supplementary material for: The Implicit Contribution of Fine Motor Skills to Mathematical Insight in Early Childhood
Source: Front Psychol. 2020 Jun 3;11:1143. doi: 10.3389/fpsyg.2020.01143 (PMC7283516; doi:10.3389/fpsyg.2020.01143)
Supplement: Supplementary file 1 [file Table_1.DOCX]

Supplementary Material

# Supplementary Tables

Supplementary Table 1

Hierarchical linear regression models predicting ordinal and cardinal finger-based representations, median split age group 1 (3;0 to 4;8), N = 39.

|  | Ordinal finger-based representation | | | | |  | Cardinal finger-based representation | | | | |
| --- | --- | --- | --- | --- | --- | --- | --- | --- | --- | --- | --- |
| Variable | B | SE B | β | *R²* | *△R^2^* |  | B | SE B | β | *R²* | *△R^2^* |
| Step 1 |  |  |  | .135 | .135 |  |  |  |  | .190* | .190* |
| Dexterity | .519 | .230 | .394* |  |  |  | .664 | .237 | .467** |  |  |
| Graphomotor skill | -.101 | .195 | -.090 |  |  |  | -.125 | .200 | -.104 |  |  |
| Step 2 |  |  |  | .351** | .216** |  |  |  |  | .393** | .204** |
| Dexterity | .362 | .207 | .275 |  |  |  | .500 | .213 | .351* |  |  |
| Graphomotor skill | -.048 | .172 | -.043 |  |  |  | -.070 | .177 | -.058 |  |  |
| Age in months | .241 | .073 | .477** |  |  |  | .253 | .075 | .463** |  |  |
| Step 3 |  |  |  | .446** | .095 |  |  |  |  | .476** | .083 |
| Dexterity | .288 | .209 | .218 |  |  |  | .422 | .216 | .297 |  |  |
| Graphomotor skill | .014 | .171 | .012 |  |  |  | -.002 | .176 | -.002 |  |  |
| Age in months | .076 | .106 | .151 |  |  |  | .081 | .109 | .148 |  |  |
| Working memory (forward span) | .292 | .460 | .111 |  |  |  | .267 | .475 | .094 |  |  |
| Working memory (backward span) | .601 | .401 | .242 |  |  |  | .523 | .414 | .195 |  |  |
| Nonverbal intelligence | .205 | .146 | .258 |  |  |  | .247 | .151 | .288 |  |  |
| Note. ^*^ = p < .05, ^**^ = p < .01 | | | | | | | | | | | |

Supplementary Table 2

Hierarchical linear regression models predicting ordinal and cardinal finger-based representations, median split age group 2 (4;9 to 6;3), N = 41.

|  | Ordinal finger-based representation | | | | |  | Cardinal finger-based representation | | | | |
| --- | --- | --- | --- | --- | --- | --- | --- | --- | --- | --- | --- |
| Variable | B | SE B | β | *R²* | *△R^2^* |  | B | SE B | β | *R²* | *△R^2^* |
| Step 1 |  |  |  | .020 | .020 |  |  |  |  | .019 | .019 |
| Dexterity | .088 | .108 | .131 |  |  |  | .080 | .100 | .130 |  |  |
| Graphomotor skill | -.037 | .087 | -.068 |  |  |  | -.028 | .081 | -.055 |  |  |
| Step 2 |  |  |  | .110 | .090 |  |  |  |  | .116 | .098 |
| Dexterity | .105 | .105 | .157 |  |  |  | .097 | .096 | .156 |  |  |
| Graphomotor skill | -.036 | .084 | -.067 |  |  |  | -.027 | .078 | -.055 |  |  |
| Age in months | .112 | .058 | .301 |  |  |  | .108 | .053 | .314 |  |  |
| Step 3 |  |  |  | .324* | .214* |  |  |  |  | .388** | .271** |
| Dexterity | -.006 | .111 | -.008 |  |  |  | .019 | .098 | .031 |  |  |
| Graphomotor skill | -.076 | .079 | -.140 |  |  |  | -.080 | .069 | -.159 |  |  |
| Age in months | .061 | .057 | .163 |  |  |  | .049 | .050 | .142 |  |  |
| Working memory (forward span) | -.123 | .360 | -.053 |  |  |  | .255 | .316 | .119 |  |  |
| Working memory (backward span) | .493 | .200 | .441* |  |  |  | .481 | .175 | .466** |  |  |
| Nonverbal intelligence | .061 | .070 | .157 |  |  |  | .034 | .062 | .096 |  |  |
| Note. ^*^ = p < .05, ^**^ = p < .01 | | | | | | | | | | | |
